# A phylogeny of *Cichlidogyrus* spp. (Monogenea, Dactylogyridea) clarifies a host-switch between fish families and reveals an adaptive component to attachment organ morphology of this parasite genus

**DOI:** 10.1186/s13071-015-1181-y

**Published:** 2015-11-10

**Authors:** Françoise D. Messu Mandeng, Charles F. Bilong Bilong, Antoine Pariselle, Maarten P. M. Vanhove, Arnold R. Bitja Nyom, Jean-François Agnèse

**Affiliations:** Laboratory of Parasitology and Ecology, Faculty of Sciences, University of Yaoundé 1, BP 812 Yaoundé, Cameroon; Institut des Sciences de l’Évolution, IRD UMR 226, CNRS UMR 5554, Université de Montpellier, CC 63, Place Eugène Bataillon, 34095 Montpellier Cedex 05, France; Biology Department, Royal Museum for Central Africa, Leuvensesteenweg 13, B-3080 Tervuren, Belgium; Department of Botany and Zoology, Faculty of Science, Masaryk University, Kotlářská 2, CZ-611 37 Brno, Czech Republic; Department of Biology, Laboratory of Biodiversity and Evolutionary Genomics, University of Leuven, Charles Debériotstraat 32, B-3000 Leuven, Belgium; Department of Biological Sciences, University of Ngaoundéré, BP 454 Ngaoundéré, Cameroon; Present address: Department of Biological Sciences, Higher Teacher Training College, University of Yaoundé 1, P.O. Box 47, Yaoundé, Cameroon; Present address: IRD, BP 1857, Yaoundé, Cameroon; Present address: Capacities for Biodiversity and Sustainable Development, Operational Directorate Natural Environment, Royal Belgian Institute of Natural Sciences, Vautierstraat 29, B-1000 Brussels, Belgium

**Keywords:** Phylogeny, Lateral transfer, *Cichlidogyrus amieti*, *Aphyosemion*, Nothobranchiidae, Cichlidae, Cameroon, Africa

## Abstract

**Background:**

Parasite switches to new host species are of fundamental scientific interest and may be considered an important speciation mechanism. For numerous monogenean fish parasites, infecting different hosts is associated with morphological adaptations, in particular of the attachment organ (haptor). However, haptoral morphology in *Cichlidogyrus* spp. (Monogenea, Dactylogyridea), parasites of African cichlids, has been mainly linked to phylogenetic rather than to host constraints. Here we determined the position of *Cichlidogyrus amieti*, a parasite of species of *Aphyosemion* (Cyprinodontiformes, Nothobranchiidae) in the phylogeny of its congeners in order to infer its origin and assess the morphological changes associated with host-switching events.

**Methods:**

The DNA of specimens of *C. amieti* isolated from *Aphyosemion cameronense* in Cameroon was sequenced and analyzed together with that of *Cichlidogyrus* spp. from cichlid hosts. In order to highlight the influence of the lateral transfer of *C. amieti* on the haptoral sclerotised parts we performed a Principal Component Analysis (PCA) to compare the attachment organ structure of *C. amieti* to that of congeners infecting cichlids.

**Results:**

*Cichlidogyrus amieti* was found to be nested within a strongly supported clade of species described from *Hemichromis* spp. (i.e. *C. longicirrus* and *C. dracolemma*). This clade is located at a derived position of the tree, suggesting that *C. amieti* transferred from cichlids to Cyprinodontiformes and not inversely. The morphological similarity between features of their copulatory organs suggested that *C. amieti* shares a recent ancestor with *C. dracolemma*. It also indicates that in this case, these organs do not seem subjected to strong divergent selection pressure. On the other hand, there are substantial differences in haptoral morphology between *C. amieti* and all of its closely related congeners described from *Hemichromis* spp..

**Conclusions:**

Our study provides new evidence supporting the hypothesis of the adaptive nature of haptor morphology. It demonstrates this adaptive component for the first time within *Cichlidogyrus*, the attachment organs of which were usually considered to be mainly phylogenetically constrained.

## Background

Teleost fishes of the order Cyprinodontiformes, commonly called cyprinodonts, or rivulines, livebearers and killifishes [1–3], are well known ornamental fishes. American representatives like xiphos (*Xiphophorus* Heckel, 1848) and guppies (*Poecilia* Bloch & Schneider, 1801) have been adopted as model species featuring in an increasing number of laboratory studies [4–6]. This is also the case for some African representatives, such as species belonging to *Nothobranchius* Peters, 1868 [7–10]. They are also established models in ecology and evolutionary biology [11–16] and parasitology [17, 18]. Evolutionary-parasitological research on these fishes often deals with monogeneans, a species-rich clade of mostly ectoparasitic flatworms. Fish-monogenean systems are established models to study the evolution of host-parasite interactions (e.g. [19, 20]). A diverse fauna of gyrodactylid monogeneans has been described from cyprinodontiform hosts in both the Neotropics [21] and Africa [22]. The first dactylogyridean monogenean parasites from African cyprinodonts were described by Birgi and Euzet [23] on the gills of some species of *Aphyosemion* Myers, 1924 sampled in different localities [Kala, Zamakoe and Yaoundé (Central Region)] in Cameroon. Members of this fish genus in general inhabit narrow, shallow and slowly-flowing forest streams [3, 24]. One of these killifish monogenean species, *Cichlidogyrus amieti* Birgi & Euzet [23], was isolated from the gills of *Aphyosemion cameronense* (Boulenger, 1903) and *Aphyosemion obscurum* (Ahl, 1924), two related species [2]. This discovery raised questions regarding the specificity of species belonging to *Cichlidogyrus* Paperna [25]. Indeed, no representative of *Cichlidogyrus* had, at that time, ever been collected from a fish not belonging to Cichlidae [26]. Birgi and Euzet [23] therefore hypothesized that the presence of *C. amieti* on the above mentioned two African cyprinodonts was probably the result of a lateral transfer from cichlid fishes. Switches to new host species represent a substantial risk to, e.g., aquaculture and fisheries [27, 28]. They are also of fundamental scientific interest [20], e.g. in understanding disease transmission [29], host biogeography [30, 31] and the relationship between niche specialization and host range [32]. Several analyses on phylogeny and evolution of host specificity of the monogenean gill parasites of African cichlids have been conducted [33–36]. However, congeners infecting non‐cichlids such as *Cichlidogyrus amieti* have not yet been included in these analyses. Hence the aspect of host-switching over larger phylogenetic distances was not looked into. Moreover, Pariselle et al. [19, 31] raised the question of the origin of *Cichlidogyrus* spp. described from cichlid hosts in Africa. Based on fossil, genetic and parasitic evidence, the authors hypothesized that cichlids may have originated from Madagascar [37, 38] after the Gondwanan split and subsequently dispersed over Africa, Central and South America, India and the Middle East across various marine pathways [31, 38–40]. In this case, these teleosts would have encountered salinities that resulted in the loss of their ectoparasitic monogeneans (probably representatives of Malagasy *Insulacleidus* Rakotofiringa & Euzet [41] or one of their ancestors) which show a poor tolerance to salinity and osmotic variations [31]. It is then likely that cichlids, after reaching the African continent, have been newly colonized by an ancestor species of *Cichlidogyrus*, presumably transferred from a currently unspecified African fish. From there, the ancestor of *Cichlidogyrus* evolved and specialized on members of Cichlidae [26], and became host-specific (i.e. oioxenous [42]). As *C. amieti* is known to infect representatives of Cyprinodontiformes, it could be possible that these fish represent the origin of the first host-switch to cichlids from which the present-day species-rich assemblage of *Cichlidogyrus* spp. on old world cichlids arose. Indeed, similar radiation episodes following a switch to a new host family have been identified in monogeneans, for example in *Gyrodactylus* [43]. In gyrodactylids, host-switching is even considered an important speciation mechanism [44]. It has been suggested for a range of monogeneans that colonization of different hosts is associated with morphological adaptations, in particular to the attachment organ ([45] and references therein). However, morphological analysis linked the structure of haptoral hard parts in *Cichlidogyrus* to phylogenetic rather than to host-related constraints [46]. Parasites belonging to *Cichlidogyrus* infecting non-cichlid hosts have never been taken into account in this context. Therefore, the influence of phylogenetically distant host-switches on haptoral morphology and speciation of *Cichlidogyrus* remains to be tested.

This paper therefore aims at determining the position of *C. amieti* in the phylogenetic tree of *Cichlidogyrus* spp. using molecular analyses. This will allow testing whether the putative switch between cyprinodonts and cichlids happened early in the history of *Cichlidogyrus*, seeding its radiation, or whether it rather represents a more recent event. If *C. amieti* is phylogenetically close to the species that first host-switched onto a cichlid, it should be situated close to the root of the tree of *Cichlidogyrus* spp. If *C. amieti* (or its ancestor) originated from a lateral transfer from a cichlid species, it should be closely related to a species of *Cichlidogyrus* found on that cichlid.

Determining the origin of *C. amieti* will also allow us to compare it morphologically to its closely related congeners, hence assessing the changes associated with a host-switch between fish families.

## Methods

### Sample collection and PCR amplification

Specimens of *Aphyosemion* spp. from some forest streams of the central and southern plateau and the littoral plain of Cameroon were caught using a dipnet of 2 mm x 2 mm mesh size, and immediately transferred into an empty container for freezing or into 96° alcohol for fixation and conservation. In the laboratory, fishes were dissected; gills from both sides were removed, placed in glass Petri dishes and examined under a Wild dissecting microscope. Fish identifications were done following Amiet [2] and Sonnenberg [47]. The studied specimens of *Cichlidogyrus amieti* were collected from the gills of *A. obscurum* captured in the locality of Mbalelon (03°33’54”N, 011°22’07”E, 695 m), *A. cameronense* from the localities of Oman II (03°37’45”N, 011°27’40”E, 720 m), Nkol Ngbwa (02°56’53”N, 011°50’07”E, 693 m) and Nkong (03°32’58”N, 011°25’00”E, 700 m) and *A. exiguum* from Nkong. They were individually placed in-between slide and coverslip, in a drop of water and examined under a Leica DM2500 microscope equipped with a LEICA DFC425 video camera. Parasite identification was performed using the morphology and size of sclerotized parts of the attachment apparatus (haptor) and that of the genitalia (vagina and male copulatory organs) following the original description of Birgi and Euzet [23]. While some individuals were fixed and mounted in a mixture of glycerin and ammonium picrate [48] for further morphological study, three adult specimens (fixed alive together with the host and preserved in alcohol) were prepared for PCR amplification following the protocol of Marchiori et al. [49], i.e., directly without DNA extraction. Standard PCR was performed with two primers specific to the D1-D2 domain of the large subunit region (LSU) of the 28S ribosomal gene: C1 (forward; 5’-ACCCGCTGAATTTAAGCAT-3’) and D2 (reverse; 5’-TGGTCCGTGTTTCAAGAC-3’) [50]. The amplification protocol began with 2 min at 93 °C for initial denaturation followed by 40 cycles of 30 s at 93 °C, 30 s at 56 °C for annealing, 1 min 30 s at 72 °C for extension, with a final 5 min extension step at 72 °C. The different reagents’ final concentrations were as follows: GoTaq Flexibuffer (Promega) 1x, MgCl_2_ 2.5 mM, PCR nucleotide mix, 0.2 nM of each DNTP, forward and reverse primers 1 μM each, GoTaq (Promega) DNA polymerase 2 U, template DNA 0.2 μg (between 1.6and 3 μl depending on the DNA extract concentration), nuclease-free water to 20 μl. Sequencing was performed using the same primers as in initial PCR amplification: C1 and D2. Purification was performed with an Agencourt® AMPure® PCR purification kit following the manufacturer’s recommendations.

### Sequence analyses

Sequences were aligned and improved manually using BioEdit version 5.09 [51]. Additional sequences obtained from GenBank were also included in the analysis (Table 1). Aligned sequences were analysed using Maximum Likelihood (ML), Maximum Parsimony (MP) and Minimum Evolution (ME) using MEGA (Molecular Evolutionary Genetics Analysis) version 5.1 [52]. Prior to analysis, an evolutionary model for ML and ME was selected by MEGA 5.1 using the Bayesian information criterion (BIC) [53]. Models with the lowest BIC scores are considered to describe the substitution pattern the best. Support for inferred clades was obtained in all three methods through non-parametric bootstrap [54] with 2000 replicates.

**Table 1:** List of monogenean species used in this study including their host species and accession numbers for the LSU 28S rDNA sequences

Parasite Species	Host Species	GenBank Accession Number
*Cichlidogyrus aegypticus* Ergens, 1981 [73]	*Tilapia guineensis* (Günther, 1862)	HQ010021
*Cichlidogyrus amieti* Birgi & Euzet, 1983 [23]	*Aphyosemion cameronense (*Boulenger, 1903)	KT945076
*Cichlidogyrus amphoratus* Pariselle & Euzet, 1996 [74]	*Tilapia guineensis* (Bleeker, 1862)	HE792772
*Cichlidogyrus arthracanthus* Paperna, 1960 [25]	*Tilapia guineensis* (Günther, 1862)	HQ010022
*Cichlidogyrus cirratus* Paperna, 1964 [76]	*Oreochromis niloticus* (Linnaeus, 1758)	HE792773
*Cichlidogyrus cubitus* Dossou, 1982 [71]	*Tilapia guineensis* (Günther, 1862)	HQ010037
*Cichlidogyrus digitatus* Dossou, 1982 [71]	*Tilapia guineensis* (Günther, 1862)	HQ010023
*Cichlidogyrus douellouae* Pariselle, Bilong & Euzet, 2003 [72]	*Sarotherodon galilaeus* (Linnaeus, 1758)	HE792774
*Cichlidogyrus dracolemma* Řehulková, Mendlová & Šimková, 2013 [63]	*Hemichromis letourneuxi* Sauvage, 1880	HQ010027
*Cichlidogyrus ergensi* Dossou, 1982 [71]	*Tilapia guineensis* (Günther, 1862)	HQ010038
*Cichlidogyrus falcifer* Dossou & Birgi, 1984 [60]	*Hemichromis fasciatus* Peters, 1857	HQ010024
*Cichlidogyrus halli* (Price & Kirk, 1967) [77]	*Sarotherodon galilaeus* (Linnaeus, 1758)	HQ010025
*Cichlidogyrus longicirrus* Paperna, 1965 [61]	*Hemichromis fasciatus* Peters, 1857	HQ010026
*Cichlidogyrus njinei* Pariselle, Bilong Bilong & Euzet, 2003 [72]	*Sarotherodon galilaeus* (Linnaeus, 1758)	HE792775
*Cichlidogyrus pouyaudi* Pariselle & Euzet, 1994 [70]	*Tylochromis intermedius* (Boulenger, 1916)	HQ010039
*Cichlidogyrus sclerosus* Paperna & Thurston, 1969 [75]	*Oreochromis niloticus* (Linnaeus, 1758)	DQ157660
*Cichlidogyrus tiberianus* Paperna, 1960 [25]	*Tilapia guineensis* (Bleeker, 1862)	HE792776
*Cichlidogyrus yanni* Pariselle & Euzet, 1996 [74]	*Tilapia guineensis* (Bleeker, 1862)	HE792777
*Haliotrema cromileptis* Young, 1968 [64]	*Epinephelus coioides* (Hamilton, 1822)	EU523146.1
*Haliotrema johnstoni* Bychowsky & Nagibina, 1970 [65]	*Upeneus luzonius* Jordan & Seale, 1907	DQ157664.1
*Ligophorus chabaudi* Euzet & Suriano, 1977 [66]	*Mugil cephalus* Linnaeus, 1758	JN996833.1
*Ligophorus cephali* Rubtsova et al., 2006 [67]	*Mugil cephalus* Linnaeus, 1758	JN996830.1
*Thaparocleidus asoti* (Yamaguti, 1937 [68])	*Parasilurus asotus* (Linnaeus, 1758)	DQ157669.1
*Tetrancistrum* sp.	*Siganus fuscescens* (Houttuyn, 1782)	AF026114

### Principal Component Analysis (PCA)

A PCA, using Statistica 9, was performed with “standardised” measurements to avoid morphometrical differences possibly due to developmental stage of the examined parasite or the influence of temperature on the size of the sclerites [55, 56]: i.e. the length of all sclerotized haptoral parts were divided by that of uncinuli pair II (= pair V sensu Mizelle [57]), which is supposed to keep its larval size (see [58]). The following characters were used in this analysis: total length of uncinuli I [I], III [VI], IV [VII], V [IV], VI [III], VII [II]; dorsal transverse bar: total length, maximum width, distance between auricles and auricle length; ventral transverse bar: branch total length and maximum width; total length of (ventral and dorsal) anchor, and the length of their blade, shaft, guard and point. Ten specimens of each of the following species of *Cichlidogyrus* were considered: *C.* cf. *bychowskii* (Markevich [59]) (see remark below) collected on the gills of an *Hemichromis bimaculatus* Gill, 1862 (MRAC 74155-63 voucher specimen) from the Congo River at Bokalakala (2°08'00"S, 16°22'00"E) in the Democratic Republic of Congo; *C. euzeti* Dossou & Birgi [60] and *C. longicirrus* Paperna [61] on *H.* cf. *elongatus* from a small stream near Idenao (4°13’24”N, 8°59’18”E) (both) and Soo River on the road between Abang and Adjap (3°19’21”N, 11°28’55”E) and Ossa Lake near Dizangué (3°46’43”N, 10°00’02”E) (respectively) in Cameroon; *C. falcifer* Dossou & Birgi [60] on *H. fasciatus* Peters, 1852 from Banjul on the Casamance River in the Gambia (13°26’51”N, 16°35’09”W); *C. sanseoi* Pariselle & Euzet [62] and *C. teugelsi* Pariselle & Euzet [62] both on *H. fasciatus* from a small stream near Kounoukou (4°49'37"N, 6°24'04"W) (misspelled Kounougou in the original description) in Ivory Coast. The voucher specimen of *C. amieti* we deposited in the invertebrate collection of the Royal Museum for Central Africa (Tervuren, Belgium) (MRAC 37784, host: *A. cameronense*, locality: Nkol Ngbwa) was used for supplementary observations.

### Ethical approval

Fish were handled in respect with the Cameroon National Ethical Committee Reg. Num. FWAIRD 0001954.

### Remark

Paperna [61] found and re-described on *Hemichromis bimaculatus* in southern Ghana, a species of *Cichlidogyrus* he named *C. bychowskii* only based on haptoral sclerotized parts morphology. Due to the fact that this was the only species already described on this cichlid, that Paperna did not know the morphology of its copulatory organ (no drawing in the original description and description done in Russian [59]), that the haptoral sclerotized parts are quite similar in all *Cichlidogyrus* spp. from hosts belonging to *Hemichromis*, and according to Řehulková et al. [63], we think that Paperna [61] confused the species of *Cichlidogyrus* living in Africa (Ghana) on *H. bimaculatus* with *C. bychowskii* described from a dead fish from the Leningrad aquarium [59]. The latter parasite, which possesses a spirally coiled copulatory organ [63], has never been recovered from *H. bimaculatus* nor on the closely related *H. letourneuxi* in the wild in Africa. Then we consider that either Markevich’ identification of the host was wrong, or the parasite he described was laterally transferred from another cichlid host present in that aquarium. Consequently, *C. bychowskii*, of which neither type nor voucher specimens have been deposited in any museum, should be considered as a *numen nudum*. In this study the parasite species collected from *H. bimaculatus*, although morphologically related to *C. dracolemma* Řehulková et al., [63], does not necessarily belong to the latter parasite species which was described from *H. letourneuxi*. Pending genetic comparison, we therefore used *C.* cf. *bychowskii* to designate the parasites we collected from *H. bimaculatus* from the Congo River basin.

## Results

Eleven species of *Aphyosemion* Myers, 1924 (Cyprinodontiformes, Nothobranchiidae) were captured: *Aphyosemion loennbergii* (Boulenger, 1903) (266 specimens), *A. koungueense* (Sonnenberg, 2007) (5 specimens), *A. omega* (Sonnenberg, 2007) (85 specimens), *A. riggenbachi* (Ahl, 1924) (18 specimens), *A. ahli* Myers, 1933 (86 specimens), *A. raddai* Scheel, 1975 (83 specimens), *A. exiguum* (Boulenger, 1911) (100 specimens), *A. amoenum* Radda & Pürzl, 1976 (71 specimens), *A. obscurum* (46 specimens), *A. cameronense* (133 specimens) and *A. batesii* (Boulenger, 1911) (61 specimens). The parasite *Cichlidogyrus amieti* was recovered from the gills of only three of them: *A. obscurum* captured in the locality of Mbalelon (2 worms), *A. cameronense* from the localities of Oman II (2 worms) and Nkol Ngbwa (23 worms), and *A. exiguum* from Nkong (3 worms). This is the first record of *C. amieti* on *A. exiguum*.

### Phylogenetic analysis

A 827 base pair alignment for the 28S rDNA region of the nuclear genome was obtained after trimming the ends of each sequence. The three newly sequenced specimens of *C. amieti* have the same haplotype (GenBank accession number KT945076). This unique sequence was then aligned and compared to 17 other *Cichlidogyrus* sequences available in GenBank (Table 1). Sequences from other dactylogyridean representatives, namely *Tetrancistrum* sp., *Haliotrema cromileptis* Young [64], *H. johnstoni* Bychowsky & Nagibina [65], *Ligophorus chabaudi* Euzet & Suriano [66], *L. cephali* Rubtsova et al. [67] and *Thaparocleidus asoti* (Yamaguti [68]) (Table 1), were used to root the tree.

**Fig. 1:**
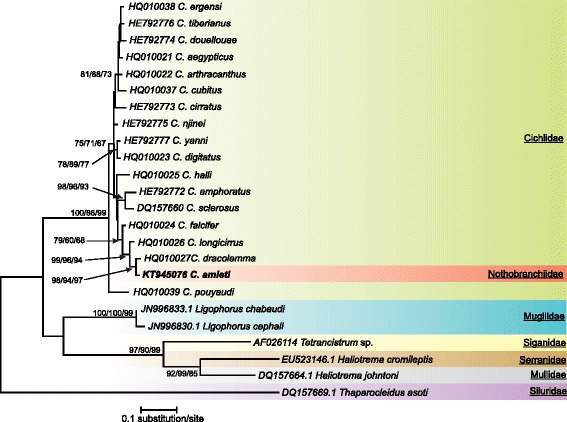
Consensus tree obtained with Maximum Likelihood analysis. Bootstrap values correspond to ME/MP/ML values respectively after 2000 iterations. Only values ≥ 50 have been indicated. Species newly sequenced for this study is in bold. Species belonging to *Ligophorus*, *Tetrancistrum*, *Haliotrema* and *Thaparocleidus* were used as outgroups. GenBank sequence ID precedes species name

A total of 445 variable sites were identified in the dataset, 327 of which were parsimony informative (i.e. shared by at least two different sequences). The optimal model of sequence evolution was TN93 + G [69]. The G parameter indicates that non-uniformity of evolutionary rates among sites is modeled by using a discrete Gamma distribution. This model was used for the subsequent analysis. The three different methods used gave congruent results summarized in Fig. 1.

Relative to the outgroup taxa, all the species of *Cichlidogyrus* appeared grouped in a monophyletic assemblage supported by high bootstrap values (100, 86 and 99 % for ME, MP and ML respectively). *Cichlidogyrus pouyaudi* Pariselle & Euzet [70] occupied a basal position in this group (bootstrap values, 75, 71 and 67 %) being the sister species of all the other species of *Cichlidogyrus* as already observed by Mendlová et al. [35].

Four clusters with high bootstrap support were apparent. One cluster was made up of *C. ergensi* Dossou [71], *C. tiberianus* Paperna [25], *C. douellouae* Pariselle, Bilong & Euzet, [72], *C. aegypticus* Ergens [73], *C. arthracanthus* Paperna [25] and *C. cubitus* Dossou [71] (bootstrap values 81, 88 and 73 %). Another cluster was made up of *C. yanni* Pariselle & Euzet [74] and *C. digitatus* Dossou [71] (78, 79 and 77 %), a third one of *C. amphoratus* Pariselle & Euzet [74] and *C. sclerosus* Paperna & Thurston, [75] (98, 96 and 93 %) and the last one of *C. falcifer*, *C. longicirrus*, *C. dracolemma* and *C. amieti*. Within this last cluster, *C. falcifer* was the sister species of *C. longicirrus*, *C. dracolemma* and *C. amieti* (99, 96 and 94 %) while *C. longicirrus* was sister to *C. dracolemma* and *C. amieti* (98, 94 and 97 %). These four clusters were not supported by high bootstrap values. Three other species: *C. cirratus* Paperna [76], *C. njinei* Pariselle, Bilong & Euzet [72] and *C. halli* Price & Kirk [77] did not appear related to any group or species.

### Principal Component Analysis (PCA)

**Fig. 2:**
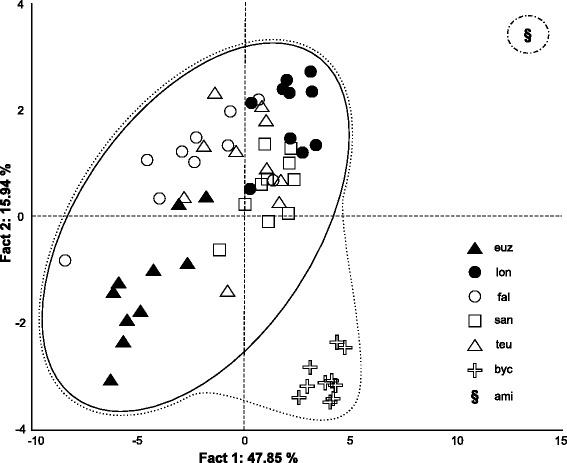
Principal component analysis scatterplot of 10 *Cichlidogyrus* specimens of each of the following species. (euz) *C. euzeti* and (lon) *C. longicirrus* both from *Hemichromis* cf. *fasciatus* in Cameroun; (fal) *C. falcifer*, (san) *C. sanseoi* and (teu) *C. teugelsi* all from *H. fasciatus* in Senegal (fal) or Ivory Coast (san and teu); (byc) *C.* cf. *bychowskii* from *H. bimaculatus* in DRC; (ami) one specimen of *C. amieti* from *Aphyosemion cameronense* in Cameroon was used for supplementary observations

The PCA analysis shows a well-defined clustering (64 % of variance on axes 1 and 2) of parasite individuals according to their respective host species (Fig. 2). The specimens of *C.* cf. *bychowskii* from *H. bimaculatus* are closer to those from *H. fasciatus s. l*. (*C. euzeti*, *C. falcifer*, *C. longicirrus*, *C. sanseoi* and *C. teugelsi*) than to the one collected from *Aphyosemion cameronense* (*C. amieti*), the latter been set apart regarding the two axes. The most represented variables and their coordinates on axis 1 are DA a (–0.95), DA b (–0.93), VA a (–0.93), VB x (–0.92) and I (–0.87); and VII [II] (–0.82), VI [III] (–0.75), III [VI] (–0.70) on factor axis 2 (Table 2).

**Table 2:** Loadings and explained variance of the first two PC of the PCA conducted on the “standardized” size of sclerites

	Fact. 1	Fact. 2
Variance (%)	47.85	15.94
I (I)	−0.873132	−0.389889
III (VI)	−0.535295	−0.709933
IV (VII)	−0.352651	−0.638280
V (IV)	0.032798	−0.186986
VI (III)	−0.242397	−0.753181
VII (II)	−0.383708	−0.825743
DB L	−0.805421	−0.199626
DB y	−0.705113	−0.021445
DB w	−0.838653	0.044545
DB h	−0.797425	0.231792
DA a	−0.954211	0.212423
b	−0.935548	0.165900
c	−0.700350	0.015457
d	−0.864610	0.324455
e	−0.416138	0.547829
VB x	−0.926706	0.077698
VB w	−0.790515	0.033730
VA a	−0.931174	−0.004236
b	−0.933118	0.063475
c	−0.532477	−0.117323
d	−0.720919	0.190484
e	−0.586523	0.488463

(I) [I], (III) [VI], (IV) [VII], (V) [IV], (VI) [III], (VII) [II] total length of uncinuli [Mizelle [57] nomenclature]; dorsal transverse bar: (DB L) total length, (DB y) distance between auricles, (DB w) maximum width, (DB h) auricle length; (DA a) total length of dorsal anchor:, (b) blade length, (c) shaft length, (d) guard length, (e) point length; ventral transverse bar: (VB a) branch total length, (VB x) maximum width; (VA a) ventral anchor total length, (b) blade length, (c) shaft length, (d) guard length, (e) point length

## Discussion

### Origin and host range of *Cichlidogyrus amieti*

*Cichlidogyrus* is the most species-rich ectoparasitic dactylogyridean monogenean genus known to parasitize African cichlid fishes. Species are distributed among a wide range of cichlid hosts [33, 58, 78]. The description of *C. amieti* from the gills of representatives of Cyprinodontiformes by Birgi and Euzet [23] raises the question whether a species from this fish order could have been the source host at the origin of the *Cichlidogyrus* radiation in cichlids (see theories on cichlid biogeography above)*.* An alternative explanation is lateral parasite transfer from a cichlid to a killifish host.

Our phylogenetic reconstruction indicates that *C. amieti* is phylogenetically nested within the parasites from species of *Hemichromis* Peters, 1857 at a derived position of the tree. Although we cannot rule out incomplete taxon coverage of Central West African *Cichlidogyrus*, the present results suggest that *C. amieti* results from a recent transfer from cichlids to nothobranchiids. That is in accordance with the Birgi and Euzet [23] hypothesis. Such lateral transfer or host-switch can occur between related host species [31, 33, 79], but even between phylogenetically distant host species, both in artificial and natural conditions [19, 20, 80–84].

*Aphyosemion* spp. inhabit small forest streams [2, 3] where they live in sympatry with *Hemichromis* spp.. Bilong Bilong [85], based on morphological features, already hypothesized that *C. amieti* could derive from *Hemichromis*’ monogeneans.

Birgi and Euzet [23] reported that *C. amieti* was restricted to *A. cameronense* and *A. obscurum*, two species belonging to the same lineage (i.e. the *A. cameronense* group), but differing from one another by their biology and the fact that they are never found together in the same biotope [2]. In this study, *C. amieti* was also collected from *A. exiguum*, a species that does not belong to the *A. cameronense* group. This new host record can be explained by the sympatry of *A. exiguum* and *A. cameronense* or *A. obscurum* and by the relative phylogenetic proximity of these fish species (compared to the phylogenetic distance between species of *Aphyosemion* and *Hemichromis*).

### Influence of host-switching on haptoral and reproductive morphology

**Fig. 3:**
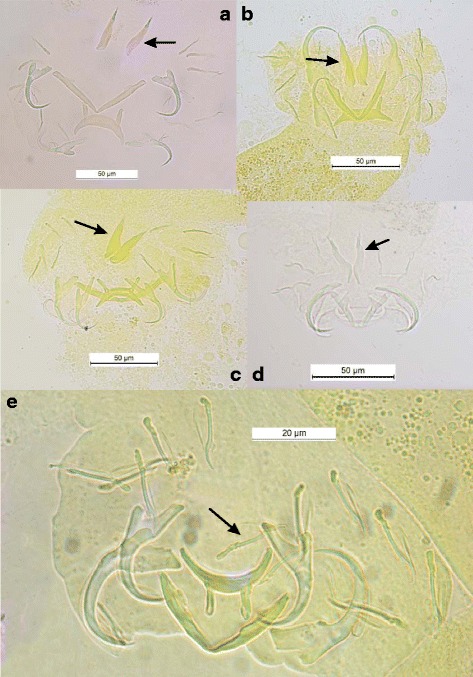
Haptoral sclerotized parts of some *Cichlidogyrus* spp. parasitizing *Hemichromis* spp. and *C. amieti* Birgi & Euzet [23] from *Aphyosemion cameronense* Boulenger, 1903. (**a**) *C. longicirrus* Dossou & Birgi [60]; (**b**) *C. euzeti* Dossou & Birgi [60]; (**c**) *C. falcifer* Dossou & Birgi [60]; (**d**) *C.* cf. *bychowskii* (Markevich [59]); (**e**) *C. amieti* Birgi & Euzet [23]. Arrow indicates uncinuli pair I [I]

**Fig. 4:**
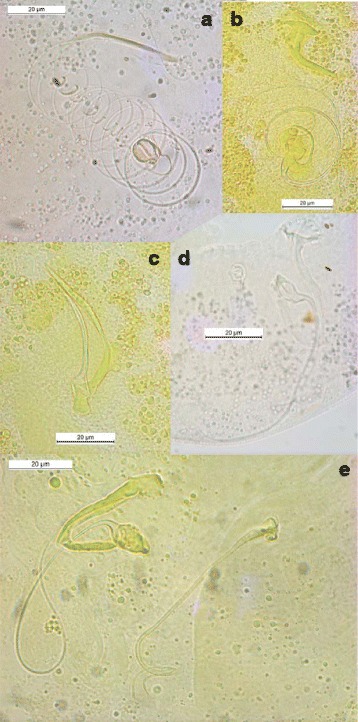
Male copulatory organs of some *Cichlidogyrus* spp. parasitizing *Hemichromis* spp. and *C. amieti* Birgi & Euzet [23] from *Aphyosemion cameronense* Boulenger, 1903. (**a**) *C. longicirrus* Dossou & Birgi [60]; (**b**) *C. euzeti* Dossou & Birgi [60]; (**c**) *C. falcifer* Dossou & Birgi [60]; (**d**) *C.* cf. *bychowskii* (Markevich [59]); (**e**) *C. amieti* Birgi & Euzet [23] (male copulatory organ on the left, vagina on the right)

While the morphology and size of the sclerotized parts of the haptor and copulatory organs of species of *Dactylogyrus* Diesing, 1850 [86], *Anacanthorus* Mizelle & Price, 1965 [87] or other genera are subject to distinct selective constraints [88–90], for *Cichlidogyrus* spp. these sclerotized parts seem to be mostly shaped by phylogenetic constraints [33, 35, 46]. In this case, the haptoral sclerite morphology is more suitable for inferring phylogenetic relationships, while the morphology of the reproductive organs is more useful for species-level identification, probably because of its faster evolutionary change [33, 35, 46]. In fact, for a given host species, the constraints on the haptoral sclerites aim to harmonize their morphologies (adapted to attach to the specific host’s gills), when those on the reproductive organs aim to make their morphologies mechanically incompatible, so profoundly different (leading to their reproductive isolation) (see Figs. 3 and 4).

Working on species of *Dactylogyrus*, Šimková et al. [91] stated that congeneric monogenean species occupying similar niches tend to have similar attachment organs. This resemblance is due to the fact that they are subject to the same considerable selective pressure imposed by the microhabitat within the host, possibly the gill morphology [92–96]. When these parasites occur on different host species, their attachment organs tend to differ from each other in their morphology and/or size, because different host species may have different gill structures. As pointed out by Šimková et al. [91], the morphology of the haptor is therefore an important adaptation of parasites to their hosts (host specificity) and to specific sites within their hosts (niche preference).

The phylogenetic tree obtained in this study (Fig. 1) suggests that *C. amieti* clusters within the monophyletic group already proposed by Mendlová et al. [34] and Řehulková et al. [63], made up of *C. longicirrus*, *C. dracolemma* and *C. falcifer*, all of them parasitizing *Hemichromis* spp.. The *Cichlidogyrus* spp. that parasitize *Hemichromis* spp. have a highly homogenous configuration of their haptoral sclerotized parts (group B in Vignon et al. [46]): very large first pair and small pairs III to VII of marginal hooks (= pairs II-III-IV and VI-VII sensu Mizelle [57]) combined with short auricles that are continuous with the dorsal surface of the dorsal transverse bar (Fig. 3a, b, c and d); this morphological relationship is also well supported by the PCA analysis (Fig. 2). In contrast, in *C. amieti* all marginal hooks are of similar small size including pair I (group A in Vignon et al. [46]) (Fig. 3e); this difference is also highlighted by our PCA results where this species is set apart regarding the two axes from all the other ones parasitizing *Hemichromis* spp. (Fig. 2). Therefore we hypothesize that, as soon as the ancestor of *C. amieti* (with group B morphology of its haptoral sclerites) colonized a species of *Aphyosemion*, selective pressures lead to a substantial morphological change in the haptoral sclerites, the most visible being the drastic reduction of the size of marginal hook pair I (Fig. 3 arrows). Vignon et al. [46], focusing on the same monogenean genus, did not find any evidence of host-related adaptation of the haptor morphology. However, these authors only considered *Cichlidogyrus* spp. infecting cichlids. The present study, considering also a more distant host-switch, provides new evidence supporting the hypothesis of the adaptive nature of haptor morphology also within *Cichlidogyrus* in accordance with studies on other monogeneans by Morand et al. [97, 98], Huyse and Volckaert [99] and Bush et al. [100].

Rohde and Hobbs [101] and Šimková et al. [91] showed that congeneric parasite species living in the same niche presented differences in the morphology or size of their reproductive organs, as a result of random differentiation, which made possible their coexistence according to the hypothesis of reinforcement of reproductive barriers by mate discrimination [102–104]. This is the case for *Cichlidogyrus* spp. harbored by *Hemichromis* spp., which are well differentiated from each other by the morphology or size of their reproductive organs (Fig. 4a, b, c and d). Regarding the male copulatory organ (MCO) of *C. amieti*, we notice that it presents a tubular filiform single-looped penis without swollen portion and with a well-developed heel, and a sharply curved accessory piece with rounded ending [23, 58] (Fig. 4e). It resembles *C. dracolemma* (Fig. 4e) as pointed out by Řehulková et al. [63]. Therefore we may assume that *C. dracolemma* or a close relative was transferred from a species of *Hemichromis* to an *Aphyosemion*. This suggestion is strongly supported by the close phylogenetic relationship between these two parasite species (Fig. 1). Finally, the specialization of these two parasite species on phylogenetically distant hosts (i.e. cichlid and killifish species) prevented their hybridization, thus explaining why their MCO morphologies have not been affected by selective pressure and thus did not substantially diverge.

## Conclusion

Phylogenetic analysis suggests that *C. amieti* results from a recent host-switch from a cichlid species belonging to *Hemichromis*. The fact that the haptoral hard parts of *C. amieti* are of a different morphotype than those of its closely related congeners infecting *Hemichromis* spp., is the first proof, within *Cichlidogyrus*, of an adaptive component to haptoral morphology influenced by transfer to a new host. Previously, haptoral morphology of *Cichlidogyrus* was considered to be mainly phylogenetically constrained. The changes in the haptoral elements after the host-switching event are in stark contrast to the similarity in male genital morphology to the parasites of representatives of *Hemichromis*. As genital differentiation between monogenean species is thought to be linked to reinforcement of parasite genetic isolation within the same host species, we suggest this similarity is a consequence of *C. amieti* having speciated as a result of host-switching. This study underscores the potential of *Cichlidogyrus* as a model to test the influence of ecology and evolution on parasite speciation [19, 78]. The fact that the adaptive component of haptoral morphology of *Cichlidogyrus* was not inferred when including only species infecting cichlids, also demonstrates the importance of including the full phylogenetic or host range of a parasite clade to reconstruct its speciation mechanisms.
